# Clinical features of depression in women and men with bipolar affective disorder

**DOI:** 10.1192/j.eurpsy.2022.1028

**Published:** 2022-09-01

**Authors:** A. Stolyarova, N. Tyuvina, V. Balabanova

**Affiliations:** Sechenov university, Psychiatry And Narcology, Moscow, Russian Federation

**Keywords:** Depression, Gender differences, Bipolar Affective Disorder

## Abstract

**Introduction:**

Knowledge of the main markers of bipolarity, clinical features and the course of depression within BPAD in men and women will contribute to the correct diagnosis, prog- nostic assessment of the disease course and administration of an adequate therapy.

**Objectives:**

The aim of the investigation was to study the clinical features of depression in men and women with BPAD in order to identify markers of bipolarity, facilitate a diagnostic search and determine therapeutic tactics.

**Methods:**

The study was conducted from 2018 to 2020 in outpatient and inpatient conditions of the S. S. Korsakov Psychiatric Clinic of Sechenov University. 100 patients (50 women and 50 men) with a diagnosis of F31.3-F31.5 according to ICD-10 were examined by the clinical method.

**Results:**

In the structure of the depressive phase in men, the following were more common: pronounced seasonality (with deterioration in autumn-winter) and daily fluctuations in the state (with improvement in the evening), anesthesia of the senses, depersonalization-derealization syndrome, decreased libido, difficulty falling asleep and increased appetite and / or body weight, comorbid depression, panic attacks and alcohol and surfactant abuse. Depression within the BPAD in women was characterized by a more frequent presence of apathy, tearfulness, self-harm, dysmorphophobic inclusions, decreased appetite.

**Conclusions:**

The revealed features of psychopathological symptoms and correlations between some characteristics and factors, taking into account gender differences, can be used as markers of bipolarity, which will allow for an earlier and more accurate diagnosis of BPAD and adequate therapy.

**Disclosure:**

No significant relationships.

